# Development of a System for Real-Time Monitoring of Pressure, Temperature, and Humidity in Casts

**DOI:** 10.3390/s19102417

**Published:** 2019-05-27

**Authors:** Chiu-Ching Tuan, Chi-Heng Lu, Yi-Chao Wu, Wen-Ling Yeh, Mei-Chuan Chen, Tsair-Fwu Lee, Yu-Jing Chen, Hsuan-Kai Kao

**Affiliations:** 1 Graduate Institute of Electronics Engineering, National Taipei University of Technology, Taipei 10608, Taiwan; cctuan@ntut.edu.tw (C.-C.T.); chiheng@cgmh.org.tw (C.-H.L.); jean25318@gmail.com (Y.-J.C.); 2 Department of Radiation Oncology, Chang Gung Memorial Hospital at Linkou, Linkou 33305, Taiwan; 3 Interdisciplinary Program of Green and Information Technology, National Taitung University, Taitung 95092, Taiwan; alanwu@nttu.edu.tw; 4 Department of Orthopedic Surgery, Chang Gung Memorial Hospital at Linkou, Linkou 33305, Taiwan; yeh610128@gmail.com (W.-L.Y.); cat0520307@cgmh.org.tw (M.-C.C.); 5 Medical Physics and Informatics Lab., Department of Electronics Engineering, National Kaohsiung University of Science and Technology, Kaohsiung 80778, Taiwan; tflee@kuas.edu.tw; 6 Bone and Joint Research Center, Chang Gung Memorial Hospital, Linkou 33305, Taiwan; 7 College of Medicine, Chang Gung University, Taoyuan 33305, Taiwan

**Keywords:** cast fixation, fractures, swathing a cast, reliable reference data, nonlinear error, human interface, soft skin cushioning, cast wrinkles, APP

## Abstract

Cast fixation is a general clinical skill used for the treatment of fractures. However, it may cause many complications due to careless treatment procedures. Currently, swathing a cast for a patient can only be determined by a doctors’ experience; however, this cannot be determined by the value of pressure, temperature, or humidity with objective and reliable equipment. When swathing a cast for a patient, the end result is often too tight or too loose. Hence, in this paper we developed a sensor for detecting pressure, temperature, and humidity, respectively. This could provide reliable reference cast data to help physicians to understand the tightness of cast swathing and to adjust the tightness of cast swathing instantly to alleviate a patient’s complications caused by excessive pressure or overheating. In this paper, six pressure sensors and one temperature–humidity sensor are used to detect the pressure, temperature, and humidity in an arm swathed with a cast to confirm whether the tightness of the cast is fixing the fracture efficiently, while avoiding causing any damage by using excessive pressure. Currently, the variation in temperature and humidity can be detected by the inflammation of the wound, displaying secretions, and fever in the cast. Based on the experiments, the voltage and power conversion coefficients of the developed sensors could be compensated for by the nonlinear error of the sensor. The experimental results could be instantly displayed on a human interface, such as a smart mobile device. The average skin pressure in a swathed cast was 12.14 g and ranged from 5.0 g to 17.5 g. A few casts exceeded 37.50 g. The abnormal pressure of wrinkles produced during swathing a cast often ranged from 22.50 g to 38.75 g. This shows that cast wrinkles cause pressure on the skin. The pressure caused by cast wrinkles on bone protrusions ranged from 56.5 g to 84.4 g. Compared to other parts that lacked soft skin cushioning, the pressure of cast wrinkles that occurred in the ulna near the protrusion of the wrist bone increased averagely. The pressure error value was less than 2%, the temperature error was less than 1%, and the humidity error was less than 5%. Therefore, they were all in line with the specifications of commercially available products. The six pressure detection points and one temperature and humidity detection point in our newly designed system can accurately measure the pressure, temperature, and humidity inside the cast, and instantly display the corresponding information by mobile APP. Doctors receive reliable reference data and are instantly able to understand the tightness of the swathed cast and adjust it at any time to avoid complications caused by pressure or overheating due to excessive pressure.

## 1. Introduction

Fractures are common accidental injuries, which often occur due to major impacts [1]. Fractures occur in a variety of age groups. For example, children with softer bones are more likely to suffer fractures from an impact compared to adults. Young adults mostly suffer from fractures related to work accidents or traffic accidents. Elderly people who have symptoms of osteoporosis can easily suffers fractures due to colliding or falling. The clinical fixation of fractures involves using a cast to fix bones [2], while some even need to be treated surgically [3]. The casts for fixing steel nails are often used both pre-surgery and post-surgery to prevent bone deformation. Yet, the fixation of casts still often causes harm to patients due to improper fitting of the cast. For example, if the cast is too tight it can cause pain, poor blood circulation, and compartment syndrome. Sometimes, it may cause the patient to need amputation [4]. However, if the cast is too loose, it will not achieve a fixed therapeutic effect. Therefore, cast fixation is an important treatment for patients with fractures.

In [5], the pressure of the cast on swollen skin was measured with different cutting methods to evaluate the decompression effect. The volunteer’s arm was fitted with a cast. A saline bag was placed on the arm, and the swelling of the affected limb was simulated by injecting air. The average pressure of the cast on the skin was 18.2 mmHg. The average maximum pressure value was 92.5 mmHg with 126 gf/cm^2^. In [6], the swathed cast was inflated to 100 mmHg to investigate the compression of the skin by the covering material.

In [7], the possibility of the rupturing of casts was measured after limb movements or surgery. It also showed how much expansion the limbs need to reach critical pressure before the cast ruptures. A 500 ml saline solution bag was placed on the forelimb of the arm, and four rolls of casts were placed on the forearm with the saline bag. The elbow was then bent at a 90° angle, and the pressure value was measured by using a pressure transducer. Moreover, the pressure in the water bag was increased by 5 mL of water each time and recorded until the pressure value reached 120 mmHg. Because swelling of the skin may vary with different dressings, severe conditions can lead to increased pressure and symptoms of neurovascular compression; furthermore, cavity syndrome causes 120 mmHg of pressure each time when the dressing ruptures. 140 mL of water was added each time.

In [8], a study was conducted on 1135 cases of swathed casts to explore the worn and torn appearance generated, as well as skin irritation or even skin infection. Different dressings were used separately, without discussing the pressure applied between the dressing and the splint. Pressure was applied to simulate the fracture of the dressing to explore the temperature of the dressings or splints on the arm. Three pressure sensors were used, as well as a thermocouple, and three different dressings were used in order to analyze the pressure and temperature. Then, the change of pressure, temperature, and humidity values in the drying, cooling, and hardening process of the dressing was observed. The experimental results suggest that pressure is an important factor leading to an increase in the temperature of the dressing.

The temperature of a cast varies depending on the temperature of the soaking water. When clinicians use a cast, a temperature of 25 °C for warm water is often used to avoid high temperatures generated from the heat release caused by the rapid solidification of the cast. Unless there are special reasons to do so, hot water is usually not used. The temperature of a cast is also related to its thickness: a thicker cast layer may engender a higher temperature. Thus, when fixing a patient with a cast it must be ensured that the cast’s cotton pad completely protects the skin from the released heat to avoid skin burns. The interval for changing a cast is about 10 min, But subsequent intervals can be differ according to the inflammation inside the cast. The humidity of a cast varies from dehydrated cast powder mixed with water to naturally dried. It takes from 24 to 48 h for plaster of Paris to dry. The synthetic gypsum (such as resin plaster) can withstand weight after 20–30 min without needing drying time. As shown in [9], temperature effect is caused by cast or fiberglass dressing materials. The authors indicated that most dressings were hardened by a patient’s reaction. The heat was generated during the hardening process, yet the probability of the released heat causing skin burn was not high. The purpose in [9] was to evaluate the effect of dressing materials on temperature changes, in which the researchers used various dressing types and temperature records for different thicknesses to change the temperature of the immersion water, evaluate external insulation factors, and control the temperature and humidity of the environment. The results showed that the material type, thickness, and water immersion temperature of the dressing were important factors for affecting the temperature. Thicker glass fibers and casts could cause temperatures to rise. The temperature of a fast-curing cast could reach 49 °C, which is a dangerous value and can increase the risk of thermal damage.

The temperature effects of dressings exerting pressure on the arm that often resulted in complications, such as burns or pressure sores, further showed that many variables affect skin temperature under the dressing, including water temperature and material quality [10]. The authors of [11,12] showed whether the skin will be burnt or not when removing a cast.

The teaching of cast fixation for new physicians is generally guided by a teacher’s subjective experience in determining the tightness of the cast, which usually lacks any scientific data reference. In [13], it was confirmed that there is a lack of objective data through oral training. Only through continuous training can the level of technology improve. The tightness of cast swathing often causes harm to patient. If the affected part is associated with inflammation or infection, it will cause an increase of the temperature of the local tissue and an increase of secretion. While the limb is covered in a cast, it is not possible to directly observe the condition of the skin. In this study, we carried out detection through six pressure detection points and one temperature–humidity detection point. The pressure detection point will detect the pressure of the bone protruding from the cast and the force applied by the clinician on the cast to determine whether the tightness of the covering reaches a fixed effect value and avoids any injury caused by compression. The temperature–humidity detection point detects the wound contained inside the cast to detect the change of temperature and humidity to assess whether the wound is producing inflammation or secretion. Then, it changes the temperature and humidity around the affected part to avoid the deterioration of the wound due to the cast swathing.

## 2. Materials and Methods

**Figure 1:**
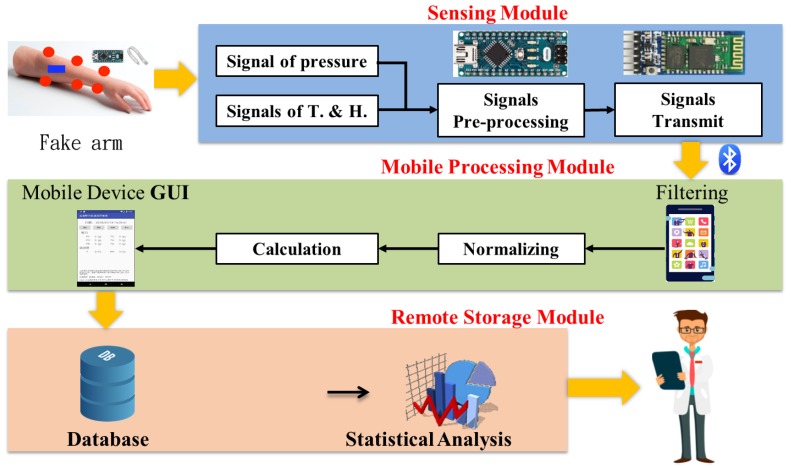
Real-time monitoring of pressure, temperature, and humidity in the cast system architecture.

In this paper, we designed a system for real-time measurement of the pressure, temperature, and humidity caused by a cast on the skin in order to provide new physicians with an objective reference value to instantly understand the influence of the casting, as simulated on a practice arm. The data in the database was also supplemented to assist the physicians in understanding the process changes in both teaching and clinical practice. The system architecture was divided into three parts. The first part was the sensing module consisting of a sensor, a development control board, and a communication device. The original pressure signal could be transmitted to the control board for calculation. The second part was the processing module used to convert the acquired data into a readable pressure value and then transmit the data to the smart mobile device via Bluetooth. The third part was the remote data storage module, which stored the received data of pressure, temperature, and humidity in the database, and transformed the meaning of numerical values into colors to display the pressure, temperature, and humidity data during the practice in real-time to provide objective reference data. The pressure sensor was placed in the area that was considered to be possibly exerting pressure. The temperature–humidity sensor was placed near the affected part. The system architecture for measuring pressure, temperature, and humidity is shown in Figure 1 [14,15].

### 2.1. Sensing Modules

**Figure 2:**
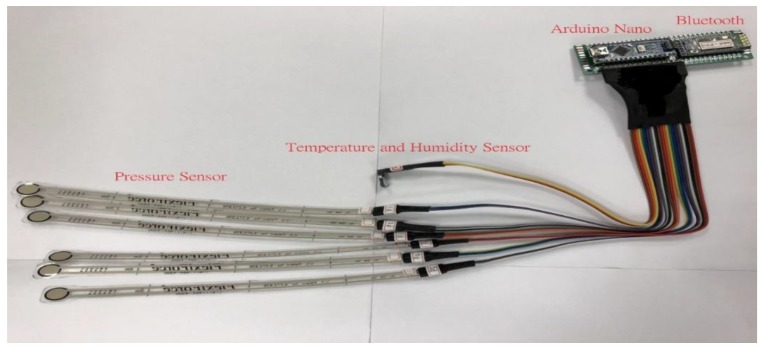
Sensing module of pressure, temperature, and humidity.

The sensing module collected the physical quantity changes of the external environment through the pressure-, temperature-, and humidity-sensing components, and sent them to the microcontroller for signal conversion. Such wireless signals were then transmitted to the handheld mobile device via the Bluetooth function. The module composition is shown in Figure 2.

The Arduino nano-embedded development board (Arduino SRL, Scarmagno, Italy) with a nano chipset of ATmega328 of a 16 MHz working clock and the internal analog-to-digital converter (ADC) resolution of 10 bits are used as the control core of the sensor module in the system. They contained 14 sets of digital and 8 sets of analog input/output pins, along with a digital I^2^C interface, which could be used to exchange data with peripheral devices. The micro controller unit (MCU) transmitted data through the I^2^C interface to the sensor, while the nano development board communicated with other devices via serial communication. The pressure sensor adopted a Flexiforce A201-1 (Tekscan Inc., Boston, MA, US) pressure sensor with a single-point sensing area diameter of approximately 9.53 mm and a pressure measurement range of 0–1 lb (approximately 0–453.6 g), under working temperature from –9 to 60 °C. The temperature–humidity sensor was SHT20 (Sensirion, Staefa, Switzerland), with a range from –40 to 125 °C, ±0.3 °C. The humidity measurement range was from 0% to 100%, ±3%. The design of this temperature and humidity sensor was small and thin in order to avoid additional compression and damage to the skin. The Bluetooth module (Guangzhou HC Information Technology Co. Ltd., Guangzhou, China) was BC417143, with supported specification of Bluetooth 2.1 + EDR with 10 meters of the communication distance. The transmission could be carried out with an operating voltage from 3.6 to 6 V and an operating temperature from −25 to 75 °C.

The detection of the pressure sensor module was through a strain effect, for which components were subjected to external force to deform the strain gauge to change the resistance value. The sensor circuit would output its electrical signal, which can be converted to units of pressure by calculation. The circuit design output value of the temperature and humidity sensor was a digital signal. The data of the measurement point can be obtained without requiring special calculation and conversion. Since the conversion curve of the sensor’s switching voltage and power obtained by the specification of the pressure sensor was nonlinear, the conversion of voltage and force with a single conversion coefficient led to a conversion error. Thus, we used the value of 18.2 mmHg, mentioned for the normal wrap, along with 92.5 mmHg as an outlier, as used in reference [5]. Through the subsequent data verification, the conversion coefficients of four different segments were obtained. The transformation coefficients of the segments were automatically determined by the program and calculated to avoid human error. The section was divided into four sections: 0–50 g, 50–200 g, 200–300 g, and more than 300 g, by the pressure conversion coefficient. The clinical measurement of this segmentation was performed when a clinician conducted a cast setting. The normal pressure value of the short arm-cast was 25 ± 5 g; the abnormal pressure value of a short arm-cast with wrinkles reached 37.5 g; and the pressure on bone protrusions tended to cause compression on the skin, reaching up to 103.9 g. The other type was the short arm-cast, with abnormal wrinkle pressure as high as 90.9 g, and the pressure at the bone protrusion as high as 382.4 g. Although the normal acceptable pressure value should have been in the range of the first segment, the accuracy of the abnormal value or the excessive force of the clinician during the touch were not able to be ignored. Therefore, the division of the conversion coefficient of the second, third, and fourth segments were performed subsequently.

### 2.2. Software

This research used the Android system to design the mobile device application (APP) and adopted the model–view–controller model (MVC) of model, view, and controller. The model was an application logical for the APP to capture and store the state of the database. The inspection was regarded as the presentation of information and the displaying components of the user interface (UI). UI was established through the model, and the controller was responsible for the user’s interaction. The MVC model enhanced the efficiency of development in a way that separated model, view, and controller to create the following four functions: Bluetooth connection, value receipt, value storage, and historical data query, where the value storage and query history data were realized through the database design to achieve the effect of data visualization [16]. The data were stored through LevelDB [17], an open-source, non-associative database (NoSQL) developed by Google Inc. Since the data could not be accessed through the remote end syntax because SQL was not used, the user was required to design the server and LevelDB in order to access the data. Only one process can access the database at a time to ensure that multiple processes cannot modify the data at the same time. Representational state transfer (REST) is a software architecture program [18]. Such master–slave architecture has an advantage of using a browser as a client (computing) to efficiently improve response speed through caching and saving resources for server operations. Web Service was designed in accordance with RESTful way, in terms of scalability, ease of use, and maintainability. RESTful is thus superior to traditional Web APIs.

### 2.3. Measuring Position

**Figure 3:**
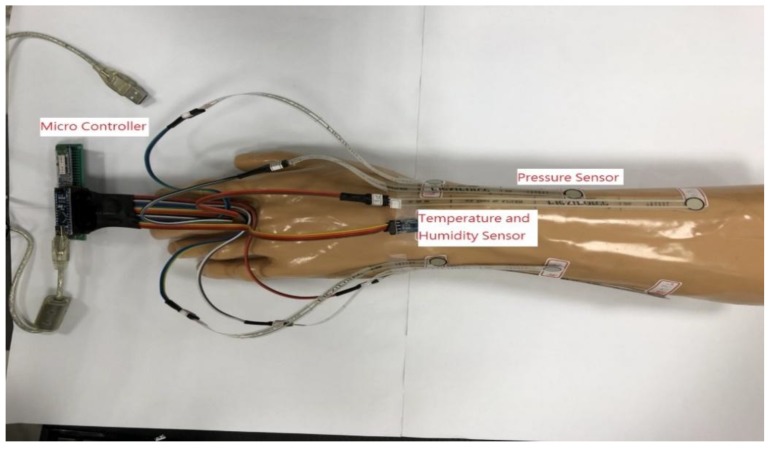
Location map of pressure, temperature, and humidity detection point on a simulated arm.

Six pressure detection points and one temperature–humidity detection point in the system were fixed to the simulated arm (Sawbones, Vashon Island, WA, USA) for detection, as shown in Figure 3. The red point was the position of the pressure detection point. The blue point was the position of the temperature–humidity detection point. There were four points on the ulna and the radius, and the other two points were placed on the radius near the ulna of the elbow; the points were indicated by code P1–P6. The temperature–humidity detection point was set near the inside of the elbow. The temperature code was represented by T, and the humidity code was represented by RH. This study used a simulated arm to carry out experiments without the volunteers and the design of measurement actions. The system was an immediate monitoring system. The measurement time varied depending on the individual cast swathing time of the clinician and the sampling frequency was 10 Hz.

## 3. Results and Discussion

### 3.1. Pressure Verification

The pressure sensor converted the piezoelectric signal caused by the affected limb with a cast to understand the pressure range caused. Under normal circumstances, the range should be from 0 to 35 g. However, it may vary from 35 g to 350 g when there is abnormal pressure. The verification range would include the pressure values for this zone, and the pressure system values were verified through standard test weight to ensure their reliability for reference. The parameters were designed in two parts. The first part was the first section of the pressure conversion section of 0–50 g. The second part was the pressure abnormality of 100–450 g. Each pressure weight parameter was measured six times. Each measurement was for 10 seconds at a measurement frequency of 10 Hz. A total of 600 data points were calculated for the average value. Then, the calculated error was performed through the calculation of the average value and the standard test weight. The experimental results showed a minimum error of 1.31% and a maximum error of 1.82% in the first segment. The minimum error of the second segment was 1.27%, and the maximum error was 1.68%. The errors of both sections were within 2%. Hence, it was confirmed that the values presented by the system were reliable.

### 3.2. Temperature and Humidity Verification

The temperature and humidity were verified by MHK-225LK (Terchy, NanTou, Taiwan). Each temperature and humidity parameter was measured six times and for 10 seconds each time. The sampling frequency was 10 Hz. Each experimental parameter had 600 data points, and the average value detected by the sensor was compared with the set point of the machine to ensure a certain accuracy in all kinds of temperature and humidity. The maximum error of the temperature verification result was 0.32%, and the minimum error was 0.06%. Compared with a general commercially available thermometer error, the error of this verification result was ±1 °C within the allowable range. The maximum error of the humidity verification result was 3.42%, and the minimum error was 0.24%. Compared with a general hygrometer, the verification result was ±5% within the allowable range. This proved that the values obtained by the temperature–humidity sensor was reliable.

### 3.3. Simulation Experiment

Our system explored two forms of short arm-cast and short arm-splint based on reference [19]. This study first practiced the swath in the laboratory and then asked the orthopedists to perform the swath according to their clinical experiences. The simulation results from the swath practiced in the laboratory are shown at Table 1. The experimental results from the orthopedists asked to perform the swath are shown at Table 2.

Values of P1 to P6 on the simulated arm were measured, respectively. P3 and P6 were normally swathed. P1 and P2 were with cast wrinkles. P4 and P5 were pressured by putting on weight. Details of the average value of each detection point are displayed in Table 1.

### 3.4. Simulation Test Results

**Figure 4:**
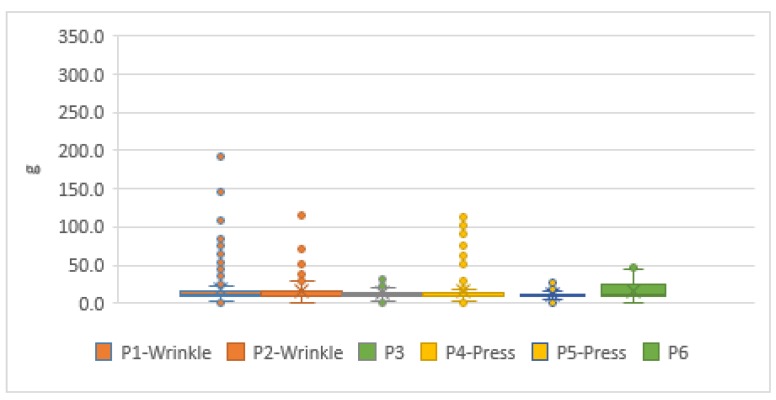
The pressure boxplot of a simulation test for a short arm swathed cast on a simulated arm.

In the laboratory, 498 pieces of information were collected from the practice of short arm-casts. Among them, the average number of P3 and P6 in a normal swath was 15.01 g. The quartile values of Q1–Q3 were 9.78–19.55 g, and the outlier values were 33.64–52.68 g, where Q1 and Q3 were the first and third quartile values. P1 and P2, which were on the cast wrinkle position, collected 498 pieces of information. The average value was 16.61 g. The quartile values of Q1–Q3 were 9.78–16.50 g, and the outlier values were 26.27–192.33 g. P4 and P5, where the pressure of weight was applied, collected 495 data. The average number was 13.62 g. The quartile values of Q1–Q3 were 9.16–12.83 g, and the outlier values were 18.33–111.72 g. The applied pressure was between 40 and 110 g, as shown in Figure 4. Limited by the factor of cast removal equipment, the measurement experiment on a simulated arm for a short arm swathed cast could not be swathed any thicker or measured for a longer time.

**Figure 5:**
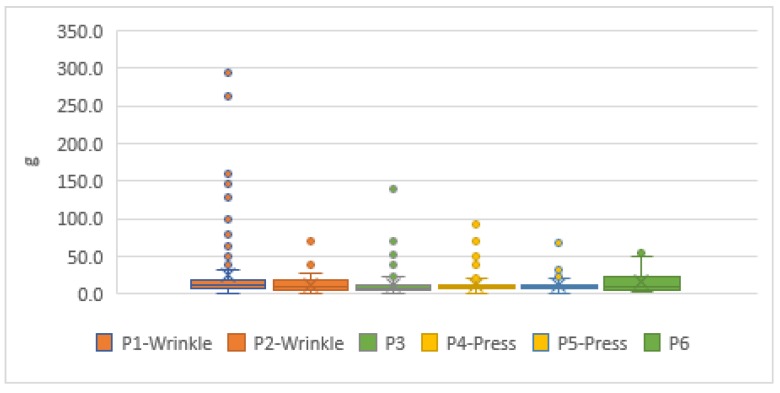
The pressure boxplot of a simulation test for short arm-cast splint on a simulated arm.

A total of 228 data points were collected from the short arm-cast splint. The average number of P3 and P6 in a normal swath was 14.05 g. The quartile values of Q1–Q3 were 9.78–19.55 g, and the outlier value was 33.64–52.68 g. P1 and P2 with cast wrinkles, as generated by a simulated swath, collected 222 pieces of information with an average number of 18.52 g, Q1–Q3 quartile values of 7.33–18.33 g, and an outlier value of 31.74–295.25 g. P4 and P5, which received pressure by applying weights, collected a total of 222 data points. The average value was 11.90 g. The quartile values of Q1–Q3 were 6.61–12.22 g. The outlier value was 20.77–92.67 g. The pressure value was 40–100 g, as shown in Figure 5.

### 3.5. Actual Test Results

A total of 185 data were collected from the orthopedists by using a short arm-cast on the simulated arm under a normal swath. An average value is considered as 12.14 g, and an average Q1–Q3 quartile value is 5.00–17.50 g. An average outlier is between 36.25 and 37.50 g. It can be seen that when the general doctor swaths the cast, the skin pressure mostly falls between 5.0 and 17.5 g, and only a minority exceeds 37.50 g. A value of 37.50 g may also be detected when the cast is smoothed by the force of hand, or in the pulling force applied when the cast is not finished. It was indicated that the pressure of the normal cast should not exceed 35 g. This was consistent with the normal range of 25 ± 5 g, as specified in the reference literature. Detailed information is shown in Table 2.

A total of 54 abnormal data points were generated during the simulation of the short arm-cast swath. The average value was 30.32 g. The quartile value of Q1–Q3 was 22.50–38.75 g. The outlier value was 53.75 g. These values showed that when the cast was swathed, the pressure mostly fell between 22.50 and 38.75 g. Generally, when the pressure value exceeds 35 g, it means that the wrinkles of the cast are causing compression on the skin. Although the pressure generated by such a swath does not affect the human skin in a short period of time, it may cause crushing of the skin after more than one month. The partial pressure, which did not exceed 35 g, means that the pressure of the wrinkles in the process did not completely exceed the warning value of compression, yet it should also be noted that excessively thick wrinkles may cause damage to the skin.

There were 301 data points on the compression of the bone protrusion during the simulation of the short arm-cast, with an average value of 77.21 g; the quartile value of Q1–Q3 was 56.49–84.42 g, and the outlier values were 123.38–168.83. The majority of the bone protrusions in the cast swathing process were 56.5–84.4 g. This was about 50 g more than the pressure value of a cast swath of the same size. This meant that the pressure here had severely oppressed the skin of the bone protrusion. The reason for the greater pressure in this site was because compared to other parts, the cast wrinkle tended to occur in the ulna near the protrusion of the wrist bone, where there is less soft skin to act as a buffer. Thus, the pressure values in this part were all greater than the normal value of 35 g.

There were 300 data points collected from the simulated wrinkles of the swathed splint, in which the average number was 81.13 g, the quartile value of Q1–Q3 was 70.13–92.21 g, and the outlier value was 124.68–128.57 g. This means that the pressure value generated by wrinkles on the skin when the splint was wrapped fell from 70.13 to 92.21 g. Since the splint was of a semi-swathed type, it generated significant pressure to force the splint to fit completely to the skin. Therefore, compared with the short arm-cast, the compression caused by the protruding wrinkles on the skin was 20 g larger. Since this range of pressure values was much higher than the warning value of 35 g, it had already caused compression on the skin. In order to avoid skin compression caused by the subsequent cast wrinkles during setting, it is particularly important to measure the required length of the splint in advance, and attention must be paid to whether the overlap site has access to the range of the cast.

**Figure 6:**
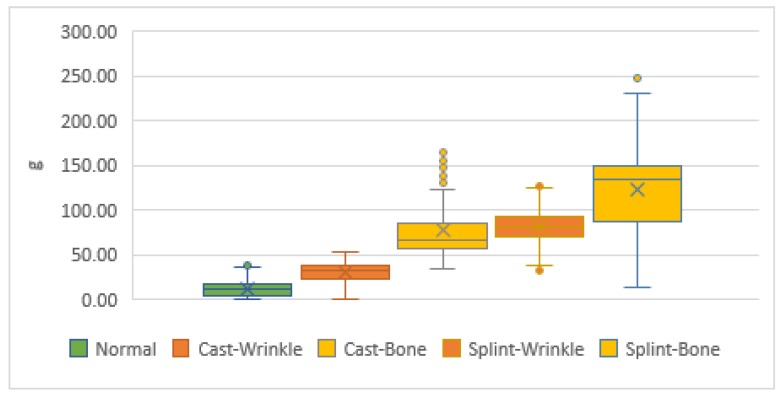
Actual test simulation of the pressure on the arm in the form of a short arm-cast and a short arm-splint.

A total of 301 pressure values on the bone protrusion generated by the simulated splint were collected, with an average value of 123.30 g, Q1–Q3 quartile value of 86.50–148.66 g, and outlier value of 229.75–247.31 g. This showed that the majority of the pressure generated by the cast splint on the bony prominence was between 86.50 and 148.66 g, which was 50 g greater than the pressure created by the cast splint on the skin. Such a pressure value might be 100 g more than that on soft skin. This was the maximum range type of a clinician-performed cast swath, whose follow-up would be provided to the clinician for reference. Although the splint is a semi-swath type, which can achieve a fixed effect as well as avoid compression when the limb is swollen, it is also easy to generate compression on the skin due to the force of the operator’s swath, as shown in Figure 6.

There are many factors that affect the temperature and humidity of the swathed cast, including the temperature of the water, the thickness of the cast, and the material used. If the water temperature is 25 °C, the temperature will be changed to 25–35 °C due to cast reaction and the hardening process time will be about 10–15 min. Under such conditions, the temperature and humidity after the hardening exothermic process will not change much. Therefore, it is necessary to use room-temperature, water-soaked cast gauze in a clinical setting to avoid the temperature of the cast being too high and causing burns. In this paper, it is shown as a pre-experiment. Hence, the experiment of temperature and humidity was verified. After institutional review board (IRB) is passed, the human experiment will be performed to discuss more experimental results. In the future, when this product is used on a patient’s arm, the changes in the wound will be able to be detected. If the wound is inflamed, it may cause an increase both in secretions and temperature. This equipment can be used to determine the change between the cast layer and the skin to provide reference data for the clinician.

Based on the existing literature of the pressure sensor verification, the average value of pressure is from 1 to 35 g in normal conditions. In abnormal conditions, the value of the pressure will be over 35 g and the maximum value of pressure will go up to 350 g. Hence, the pressure value was verified in the range of the normal and abnormal conditions to derive a parameter for the pressure values to ensure the reliability of the data generated by the pressure system in this study.

The pressure was verified by a set of weights with 500 g. The set of weights included weights of 10 g, 20 g, 50 g, 100 g, and 200 g, respectively. The number of weights with 10 g was one, the number of weights with 20g was two, the number of weights with 50 g was one, the number of weights with 100 g was two, and the number of weights with 200 g was one. Firstly, in the first section of pressure transformation the weight was from 0 to 50 g. Secondly, in the abnormal condition the weight was from 100 to 450 g. In the first section with 0–50 g, the weight parameter was measured by the coefficients of the same voltage and strength. In the second section with 100–450 g, the weight parameter was measured by the coefficients from the second section to the fourth section.

Each weight parameter was measured six times. Each time lasted for 10 seconds, and the frequency of measuring was 10 Hz. Hence, there were 100 data points for each verified parameter in each time period. Then, the average value was calculated from 600 data points at six periods. Finally, the average value subtracted from the value of weights was the difference value. In the first section, the minimal difference value was 1.31% and the maximal difference value was 1.82%. In the second section, the minimal difference value was 1.27% and the maximal difference value was 1.68%. The difference values in the first and second sections were all below 2%. Hence, the data are reliable for the clinicians to use to determine the strength of plaster fixation. These data will provide a reliable reference of the internal pressure value of wrapped plaster so clinicians can avoid relying on the clinician’s empirical judgement, since the precision within 2% meets the clinical needs. The parameter of the weights is shown at Table 3.

## 4. Conclusions

The factors of pressure that need to be changed, such as strength, wrinkles, and bone protrusions, all are problems when a cast is fixed in the fracture and the abnormal pressure value is only the change of the current measurement. It is known from experiments that the normal value is about 25 ± 5 g. If the value exceeds 35 g and has been pressing on the skin for a long time, it will cause damage to the skin. Therefore, the 35 g pressure value can be set as the warning value. When the pressure value is more than 35 g, pressure at the detection point is abnormal. At this time, the operator can further judge whether there is a wrinkle in the cast or the force at the protrusion of the bone is too large and then re-adjust the pressure of the cast swath in the simulated arm.

Whether it is a short arm-cast or a short arm-splint, the pressure applied to the bony protrusion is greater than that of the cast wrinkles pressure on the skin. It has been experimentally found that the point at which bone protrudes is more serious than the wrinkles made by the physician, though both of them have exceeded the warning value. In the process of measuring the swathed cast, the pressure of the cast swath may be increased by touching the cast or extruding the air in the cast layer.

This system had six sets of pressure detection points and one set of temperature–humidity detection points. The measuring positions were in bone protrusions where the pressure occurs. The system divided the pressure into four sections through experiments, which automatically selected the voltage and power conversion coefficients, and then displayed the measured values in real-time on the user interface of the smart mobile with the APP installed. The measurement results can be stored in a database to provide physicians with a post-inquiry record or to understand the changes in practice processes.

While the sensor is bent, its characteristics will change. In this paper, the reference of value was corrected before plaster was wrapped. Next year’s plan for fixing arms will be improved to avoid the sensor bent arbitrarily to re-evaluate performance. The characteristic curve will be discussed in the future.

## Figures and Tables

**Table 1:** Analysis of simulated experimental data using short arm-cast and short arm-splint.

	Normal Swath		Short Arm-Cast		Short Arm-Splint	
	Cast (g)	Splint (g)	Wrinkle Generated (g)	Pressure Exerted (g)	Wrinkle Generated (g)	Pressure Exerted (g)
Number	498.00	228.00	498.00	495.00	222.00	222.00
Max. value	52.68	139.65	192.33	111.72	295.25	92.67
Min. value	3.05	1.22	1.83	1.22	0.61	1.22
Range	49.63	138.43	190.50	110.50	294.64	91.45
Median	11.61	8.55	11.61	10.39	10.39	8.55
Mode	9.78	6.72	9.78	9.78	8.55	8.55
Std. deviation	8.40	15.25	16.42	13.94	32.02	11.70
Std. error	0.38	1.01	0.74	0.63	2.15	0.79
Average	15.01	14.05	16.61	13.62	18.52	11.90
Variance	70.50	232.59	269.56	194.26	1025.15	136.84

**Table 2:** Actual analysis for the utilization of a short arm-cast and a short arm-splint.

		Short Arm-Cast		Short Arm-Splint	
	Normal Swath (g)	Wrinkle Generated (g)	Bone Protrusion (g)	Wrinkle Generated (g)	Bone Protrusion (g)
Number	185.00	54.00	301.00	300.00	301.00
Max. value	37.50	53.75	168.83	128.57	247.31
Min. value	1.25	1.25	35.06	32.47	13.52
Range	36.25	52.50	133.77	96.10	233.79
Median	11.25	32.50	66.23	81.82	133.80
Mode	1.25	33.75	62.34	84.42	136.50
Std. deviation	8.35	14.60	31.02	18.06	44.05
Std. error	0.61	1.99	1.79	1.04	2.54
Average	12.14	30.32	77.21	81.13	123.30
Variance	69.65	213.04	962.07	326.04	1940.41

**Table 3:** Parameter of weights.

Weight of Weights A (g)	Average Weight by Sensor B (g)	Ratio of Difference (B − A)/A(%)
10	10.1 ± 1.4	1.31
20	20.4 ± 0.8	1.82
30	30.5 ± 0.5	1.66
40	40.4 ± 2.7	1.68
50	50.9 ± 3.4	1.82
100	101.6 ± 0.9	1.61
200	202.6 ± 4.1	1.27
300	304.2 ± 4.4	1.38
400	405.0 ± 3.8	1.25
450	455.9 ± 3.1	1.31
